# Effect of Steel-Cutting Technology on Fatigue Strength of Steel Structures: Tests and Analyses

**DOI:** 10.3390/ma14206097

**Published:** 2021-10-15

**Authors:** Sławomir Rowiński

**Affiliations:** Faculty of Civil Engineering, Wrocław University of Science and Technology, 50-370 Wrocław, Poland; slawomir.rowinski@pwr.edu.pl; Tel.: +48-71-320-2905

**Keywords:** steel structures, fatigue strength of steel, hardness, roughness, plasma cutting, water jet cutting, gas cutting, composite dowel

## Abstract

This paper presents the results of comparative fatigue tests carried out on steel S355J2N specimens cut out using different cutting methods, i.e., plasma cutting, water jet cutting, and oxyacetylene cutting. All the specimens were subjected to cyclic loading from which appropriate S-N curves were obtained. Furthermore, face-of-cut hardness and roughness measurements were carried out to determine the effect of the cutting method on the fatigue strength of the tested steel. The fatigue strength results were compared with the standard S-N fatigue curves. The fatigue strength of the specimens cut out with oxyacetylene was found to be higher than that of the specimens cut out with plasma even though the surface roughness after cutting with plasma was smaller than in the case of the other cutting technology. This was due to the significant effect of material hardening in the heat-affected zones. The test results indicate that, in comparison with the effect of the cutting technology, the surface condition of the specimens has a relatively small effect on their fatigue strength.

## 1. Introduction

Most of the steel frame structures currently built and used in, i.a., the construction industry, the marine industry, and the manufacturing industry have their parts joined together and properly shaped by, e.g., welding and cutting out from larger steel elements. Currently, oxyacetylene cutting, plasma cutting, and water jet cutting are the predominant cutting technologies. In the case of oxyacetylene cutting, computer-controlled devices usually execute the cutting line with an accuracy of 0.8–1.6 mm. The width of the slit depends on the cutting parameters, i.e., the diameter and shape of the oxygen nozzle, the cutting oxygen and inflammable gas pressure, and the cutting speed. After oxygen cutting, the cut heat-affected zone (CHAZ) is relatively wide and depends on the alloying element content in the material. In the case of low-carbon steel plates, the width of CHAZ amounts to less than 0.8 mm at the thickness of 12.5 mm and to about 3 mm at the thickness of 150 mm [1]. Plasma cutting consists in melting metal and ejecting it from the slit with a strongly concentrated electric arc flowing between a nonconsumable electrode and the workpiece. The width of CHAZ is inversely proportional to the cutting speed and depends on the composition (conductivity) of the material being cut. In the case of 25 mm thick 18-8-type austenitic steels, CHAZ is 0.08–0.13 mm wide at the cutting speed of 1.2 m/min. Water jet cutting consists in using a strongly compressed water jet formed by passing water through a small-diameter nozzle. The water jet removes the cut material from the cutting slit through erosion and cutting fatigue under high local stresses and, additionally, through micromachining when abrasive powder with a (gamet, olivine, or silica) grain size of 0.3–0.4 mm is used. The temperature of the cut edges does not exceed 100 °C (cold cutting). The cut material thickness depends on the water jet cutting parameters, i.a., the cutting speed, the water pressure, the kind and grain size of the powder, and the powder feed rate [1].

Thanks to the significant differences between the above-mentioned cutting technologies, they find application in the manufacture of all kinds of steel frame structures. The choice of a cutting technology depends on the quality requirements and the manufacturer’s technical and financial capabilities as well as on the requirements specified by the design engineer who takes into consideration the effect of the particular technologies of cutting out a steel frame structure on the latter’s ultimate and fatigue strength.

**Figure 1:**
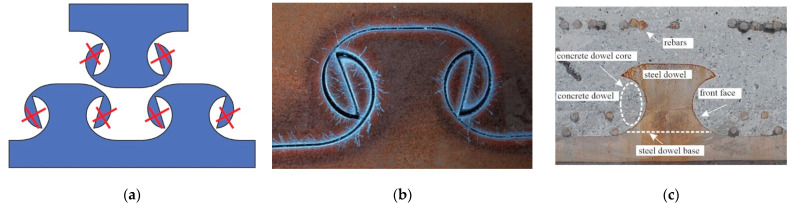
Cut-out MCL: (**a**) steel connectors with marked parts to be removed, (**b**) connector before separation of two parts of cut beam, (**c**) component parts of the innovative joint with MCL dowels.

The adverse effect of the technology of cutting out a structure on the latter’s fatigue strength was observed during extensive experimental studies on innovative connectors of the composite dowel type used in composite steel and concrete bridges [2]. Such connectors are created by appropriately joining together steel structural members and concrete. The innovative composite dowel joint is based on the idea of cutting the rolled beam’s steel web in two along a specifically shaped line (Figure 1) so that the dowels obtained in this way in each of the two parts when embedded in concrete will constitute a mechanical connector carrying the delamination forces between the steel and the concrete [3,4,5].

It was only after the PreCo-Beam [2] had been completed when the effect of the cutting technology on the load-bearing capacity of the composite dowel joint was given some thought. It was found that the roughness of the dowel’s front face after oxyacetylene cutting could be the cause of fatigue cracking in beams under cyclic loading [6,7,8].

In the literature, one can find the results of experimental studies of the effect of the technology of cutting steel on the latter’s strength. However, one should bear in mind that such studies do not take into account the complexity of this problem (particularly in the construction industry) as the experiments are conducted not on full-size models of the structures but on specimens. Moreover, the results are for the element’s particular shape, thickness, and steel grade. The effect of cutting technologies and the obtained specimen surface characteristics on the fatigue strength is described in the work of [9]. Specimens 6 and 12 mm thick with a steel strength of 240–900 MPa were tested. It was confirmed that the fatigue strength increases with the tensile strength of the steel and depending on the surface roughness. The fatigue strength of the specimens increases when their surface roughness is reduced by additional surface treatments, such as sandblasting [10]. The oxygen cut specimens have the highest fatigue strength, followed by the laser and plasma cut specimens. The gas-cut specimens have the highest surface roughness but also the highest compressive residual stress state. The plasma cut specimens have the lowest roughness, but their residual stresses are practically zero in comparison with the oxygen and laser-cut specimens [11]. The fatigue strength of plasma cut surfaces can be significantly improved with a post-cutting treatment applicable. The improvement is achieved by introducing compressive residual stress and reducing surface roughness height through grinding [12].

For steel S690Q and steel S355M, it is observed that when straight edges are cut with plasma and a laser, these cutting technologies improve, in comparison with gas cutting, the fatigue strength [13,14]. Laser-cut steel S890Q was found to have higher fatigue strength than when cut with gas or plasma [15].

It emerges from the tests carried out on specimens that the fatigue strength of steel frame structures is a complex problem sensitive to many factors. In construction, this problem is further compounded by the fact that it is not possible to directly observe the initiation and propagation of cracks (the connector is embedded in concrete) and also by the complicated interactions in the joint (delamination forces change from dowel to dowel and are transmitted through the direct pressure of the concrete against the front faces of the dowels and via the adhesive forces between the steel beam’s flat surfaces and the concrete). Therefore in order to assess the effect of the cutting technology on the fatigue strength of the material, comparative tests were carried out on dumbbell-shaped specimens cut out using different cutting technologies.

## 2. Experiment

### 2.1. Test Specimens and Test Plan

**Figure 2:**
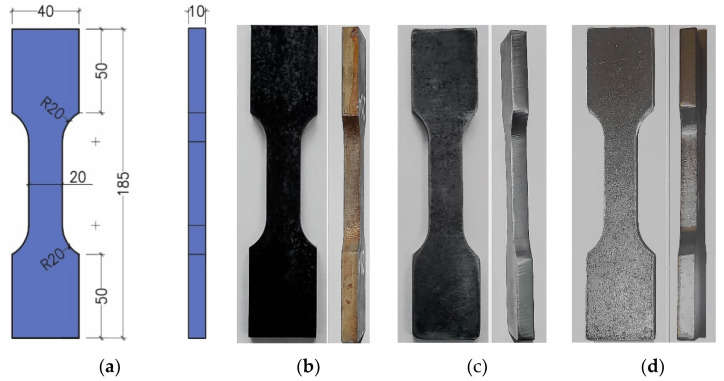
Specimen for comparative cyclic tests: (**a**) geometry (mm), (**b**) series A (water jet cut) specimen, (**c**) series B (oxyacetylene cut) specimen, (**d**) series C (plasma cut) specimen.

The specific specimen shape and dimensions (thickness and fillet radius) (Figure 2) were adopted so that the test results could apply to connectors of the composite dowel type (Figure 1). The specimens were cut out of 10 mm thick plate S355J2N along the plate rolling direction. Three series of specimens were cut out using different cutting technologies designated as: A—water jet, B—oxyacetylene, and C—plasma.

The tests were carried out on a 100 kN testing machine. The specimens were subjected to uniaxial tension-compression (R = −1) in the high cycle fatigue range until fracture. The load spectrum was sinusoidal with a frequency f = 10 Hz. Through trials, such a frequency was selected that the temperature of the specimens during tests would not exceed 60 °C. The tests were conducted in a cyclic testing machine in set stress ranges Δ*σ_i_* (Table 1) on one to four specimens for each level Δ*σ_i_*. The number of cycles N = 5 m was set as the lifespan limit. The force signal and the total deformation signal were registered during the tests. Deformation was measured along the gauge length of 25 mm by means of an extensometer.

**Figure 3:**
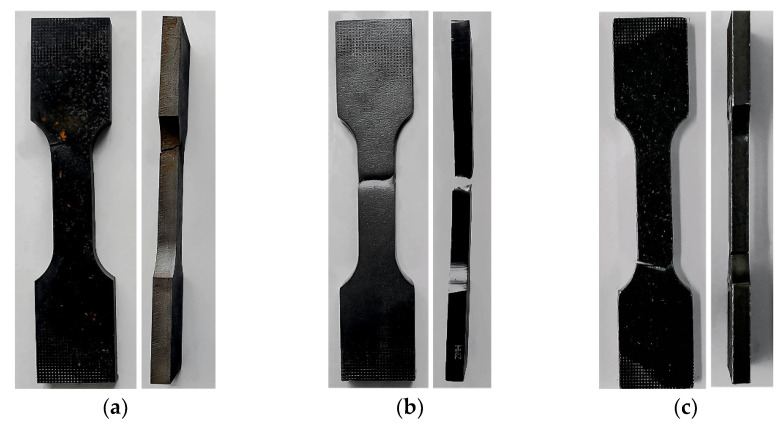
Failed specimens cut out with: (**a**) water, (**b**) oxyacetylene, (**c**) plasma.

The specimens would most often fail due to rupture in the fillet (geometric notch) area. Photographs of selected specimens after failure for each of the cutting technologies are shown below (Figure 3).

### 2.2. Results

The test results are presented in Table 2, Table 3 and Table 4.

**Figure 4:**
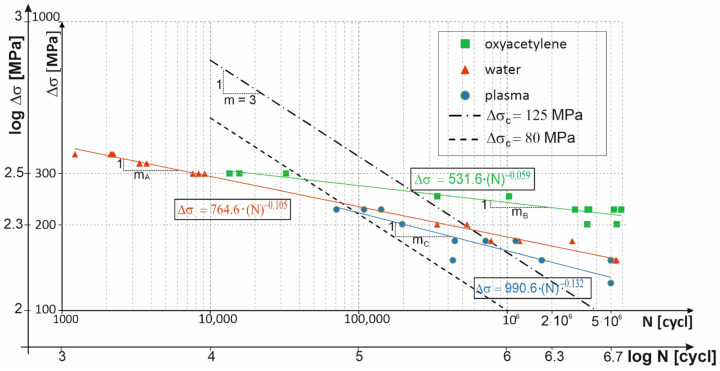
Results of tests carried out on specimens. (oxyacetylene, water, plasma).

Figure 4 shows the test results as logarithmic stress amplitude versus the logarithmic number of cycles for the three cutting technologies. Test results regression curves for each of the technologies were calculated. The standard curves for the fatigue categories of 80 MPa and 125 MPa according to standard [16] were included for comparison.

From the regression curves for the respective cutting technologies, Δ*σ_c_* stress values at N = 2 million cycles, i.e., the fatigue categories, and values of fatigue curve slope cotangent m were calculated. The results are presented in Table 5.

## 3. Cut Edge Conditions

### 3.1. Macroscopic and Microscopic Examinations of Fatigue Fractures

**Figure 5:**
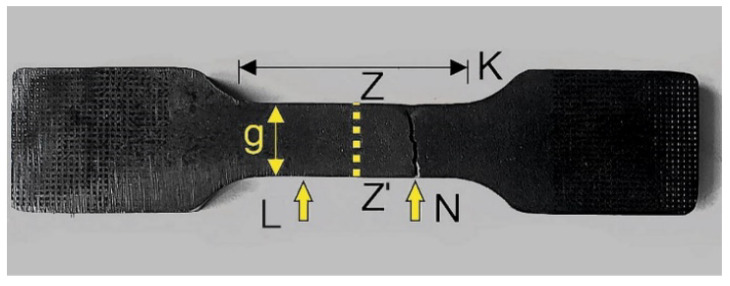
Test specimen: L—face of cut, N—fatigue fracture, K—area from which samples were taken to make metallurgical polished sections, Z-Z’—cross-sections on which polished sections were made, g—metallurgical polished section hardness measuring length.

Detailed macroscopic and microscopic analyses of selected specimens cut out with: water (2A, 14A, 17A), oxyacetylene (4B, 10B, 14B), and plasma (6C, 8C, 10C) were carried out. The specimens’ faces of cut and fatigue fracture and crack surfaces (Figure 5) were examined.

The macroscopic examinations were performed under a stereoscopic light microscope, while the microscopic examinations were carried out using a confocal laser scanning microscope and a scanning electron microscope. Samples for preparing metallurgical polished sections were cut out using a precision cutter and mounted in conductive resin. The mounted samples were ground and polished on a polishing machine and subjected to etching with 5% HNO_3_ solution.

**Figure 6:**
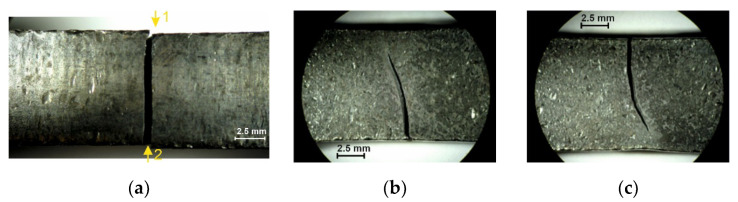
Fracture of specimen 6C: (**a**) face of cut made with plasma, (**b**) numerically denoted fracture sides no. 1, (**c**) numerically denoted fracture sides no. 2.

**Figure 7:**
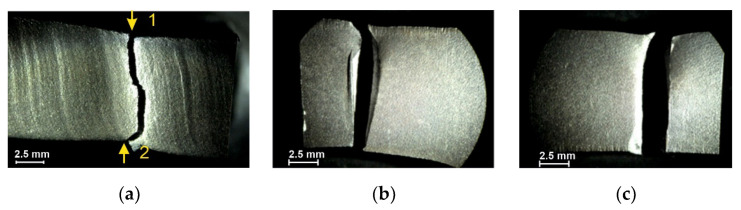
Fracture of specimen 14B: (**a**) face of cut made with gas, (**b**) numerically denoted fracture sides no. 1, (**c**) numerically denoted fracture sides no. 2.

**Figure 8:**
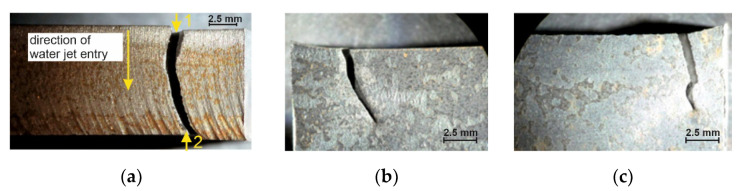
Fracture of specimen 17A: (**a**) face of cut made with water jet, (**b**) numerically denoted fracture sides no. 1, (**c**) numerically denoted fracture sides no. 2.

The tested specimens’ faces of cut showed numerous furrows resulting from cutting, along which fatigue cracks propagated (Figure 6 and Figure 7). This is particularly visible for water jet cutting, in which case the face of the cut is distinctly varied, showing an area of the entry of the water jet with an abrasive and an exit area (Figure 8).

**Figure 9:**
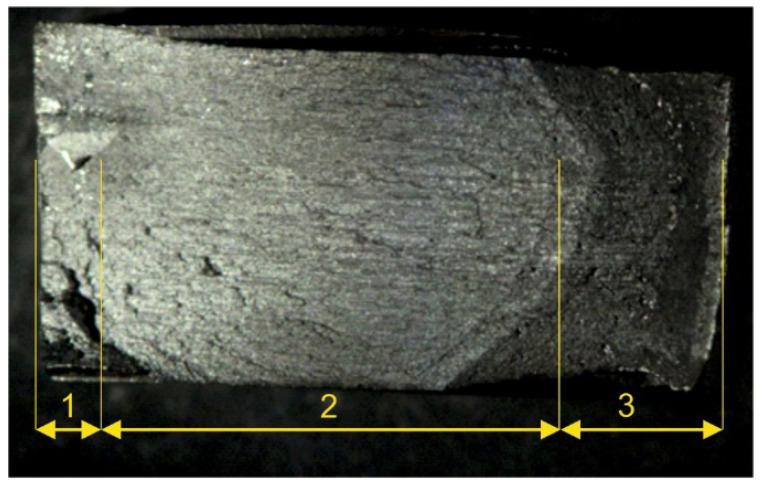
Fractured surface of specimen 14B: 1—brittle fracture area, 2—ductile fracture area, 3—granular area (end phase of fracture).

The examinations of the fractured surfaces of the specimens clearly corroborated the fatigued character of the fractures, as visible in Figure 9.

### 3.2. Investigations of Face-of-Cut Roughness

**Figure 10:**
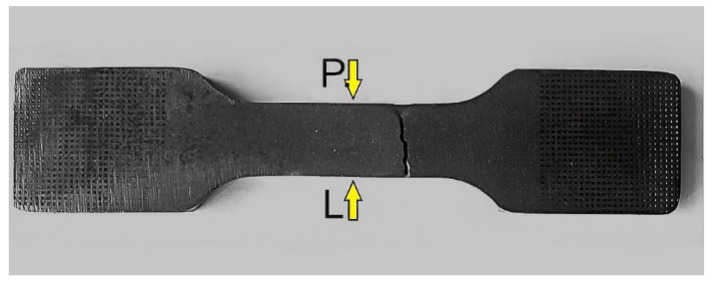
Denotations of surfaces subjected to roughness measurements.

Roughness, i.e., the arithmetic mean deviation of the profile from the mean line, on the two faces of cut denoted in Figure 10 was investigated for each specimen. The results are presented in Table 6.

### 3.3. Microscopic Examinations of Metallurgical Polished Sections

**Figure 11:**
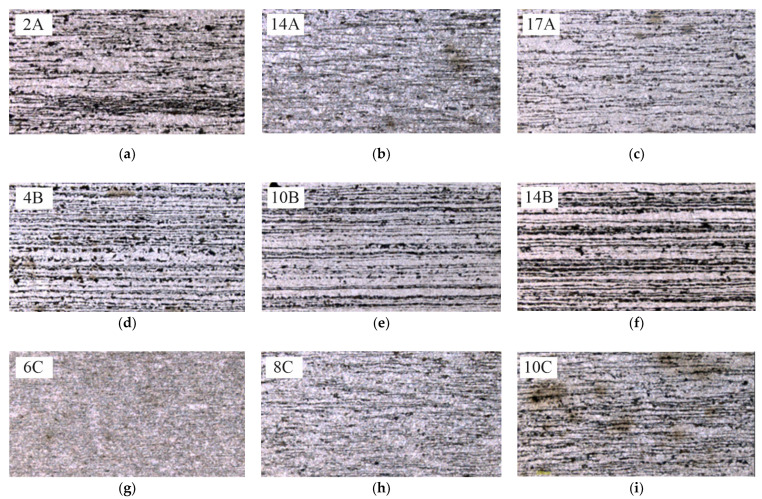
Microstructures of specimens: (**a**–**c**) cut out with water, (**d**–**f**) cut out with oxyacetylene, (**g**–**i**) cut out with plasma.

For microscopic examinations, a sample was cut out from the measurement area of the specimens consistently with the Z-Z’ plane, parallel to the fracture surface (Figure 5). After etching with 5% HNO_3_ solution, a nonequilibrium ferritic-pearlitic structure became visible (Figure 11).

### 3.4. Investigations of Cut Heat-Affected Zone

**Figure 12:**
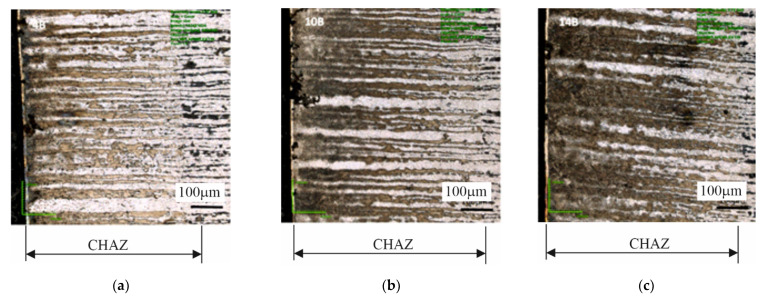
Structures in cut heat-affected zone in specimens: (**a**) 4B, (**b**) 10B, (**c**) 14B—oxyacetylene cutting technology.

**Figure 13:**
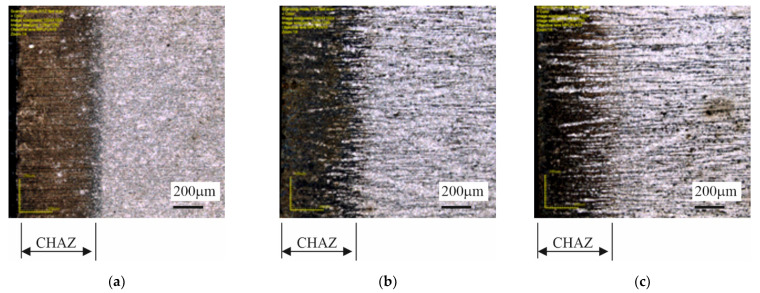
Structures in cut heat-affected zone in specimens: (**a**) 6C, (**b**) 8C, (**c**) 10C—plasma cutting technology.

**Figure 14:**
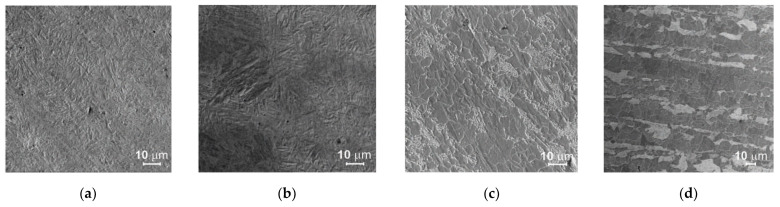
Structures: (**a**) acicular structure in CHAZ in specimens cut with plasma, (**b**) acicular structure in CHAZ in specimens cut with oxyacetylene, (**c**) pearlitic-ferritic structure in specimens cut with plasma, (**d**) pearlitic-ferritic structure in specimens cut with oxyacetylene.

The specimens cut out using the gas technology, and the plasma technology showed a distinctly changed structure at the cut edge (Figure 12 and Figure 13) due to the local heating of the material. The cut heat-affected zone (CHAZ presented in Table 7) was characterized by a martensitic structure with varied carbon content. This structure, unlike the ferritic-pearlitic structure, was more brittle and susceptible to cracking (Figure 14). No significant changes in microstructure were observed in the case of the water cut specimens.

### 3.5. Hardness Tests

Hardness was measured using the Vickers method in accordance with the standard [17]. The measurements were performed under the load of 10 kg acting over the time of 10 s.

The hardness measurements were carried out on metallurgical polished sections along segment g (Figure 5) consistent with the pearlite-ferrite banding. The distance between the cut edge and the first impression amounted to 0.3 mm. The next impressions were spaced at every 1.5 mm. In total, 10 impressions were made for each specimen.

**Figure 15:**
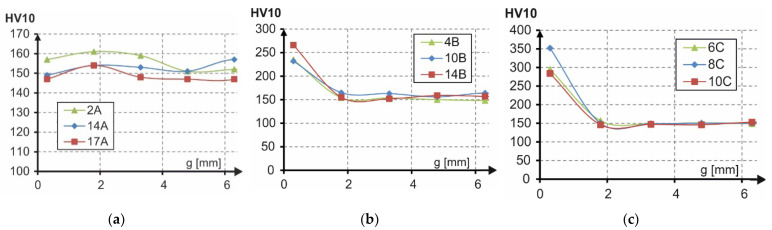
Hardness of type: (**a**) A specimens (water), (**b**) B specimens (oxyacetylene), (**c**) C specimens (plasma).

The hardness of the tested specimen materials ranged from 146 to 352 HV10 (Figure 15). Hardness values above 150 HV10 were measured exclusively at the edges of the specimens cut with oxyacetylene and plasma. The average hardness in these places amounted to 244 HV10 and 310 HV10 for the type B specimens and the type C specimens, respectively.

## 4. Analysis of Results

The specimens cut out using the oxyacetylene technology showed a longer fatigue lifespan than the other specimens cut out with plasma and water, as presented in Figure 6. Fatigue categories were calculated from the regression curves of the cutting technologies. The fatigue categories amounted to: Δ*σ_c_,_B_* = 226 MPa for oxyacetylene cutting, Δ*σ_c_,_A_* = 167 MPa for water jet cutting and Δ*σ_c_,_C_* = 146 MPa for plasma cutting. The results are above fatigue category Δ*σ_c_* = 125 MPa according to the work of [16], which is safely and most frequently adopted by building designers and applies to gas-cut metal plates with removed edge discontinuities. Unfortunately, in the case of series-produced connectors of the composite dowel type, each of the production operations, including the machining of the face of cut, is thought to add to the cost, and so efforts are made to reduce the latter by, i.a., limiting the additional treatments of the face of the cut. The current design guidelines according to the work of [16] do not take into account the effect of the cutting technology and the quality of the cut surface on the fatigue strength of the structure. Therefore it is necessary to clarify and specify more precisely the fatigue category for other cutting technologies, including water jet cutting and plasma cutting, which should have a beneficial effect on the design of steel frame structures.

In all the considered cases, the fatigue curve slope cotangents m were larger (*m_A_* = 10, *m_B_* = 17, *m_C_* = 8) in comparison with the standard curves according to the work of [16], for which *m* = 3. The slope values provide information about the speed of fracture of the specimens under variable load. In the case of oxyacetylene cutting, the specimens would fracture slowest (*m_B_* = 17), as opposed to the specimens cut out with plasma, which would fracture fastest (*m_C_* = 8). It should be noted that the results of this test depend on, i.a., the number and shape of the specimens, the character of the fatigue load, and the yield point of the material. Therefore it is difficult to directly compare the obtained results. A convergence between the results is considered to be a satisfactory outcome. In the case of steel S355, one gets curve slope *m* = 7 for oxygen cut specimens and *m =* 13 for plasma cut specimens [11], or *m =* 5.2 for plasma, *m =* 5.8 for oxygen and *m =* 16.8 for water, as described in the work of [18]. The ongoing research confirms the conservative standard recommendations for which *m* = 3 [16]. It should be added that the obtained moderate conservatism can be proper considering that small-scale specimens, in general, ensure greater fatigue reliability than large-scale beam specimens [19].

The material tests corroborated the relatively smaller surface roughness for plasma cutting (*R_a,mean_* = 0.208 μm) and oxyacetylene cutting (*R_a,mean_* = 1.444 μm) than for water jet cutting (*R_a,mean_* = 3.496 μm). The numerous furrows in the water jet entry zones in the water cut surfaces were micronotches in which the initiation of fatigue fractures would take place. It is noteworthy that the choice of cutting parameters and the thickness of the metal plate being cut have a bearing on the quality of the cut-out specimen’s surface [18].

**Figure 16:**
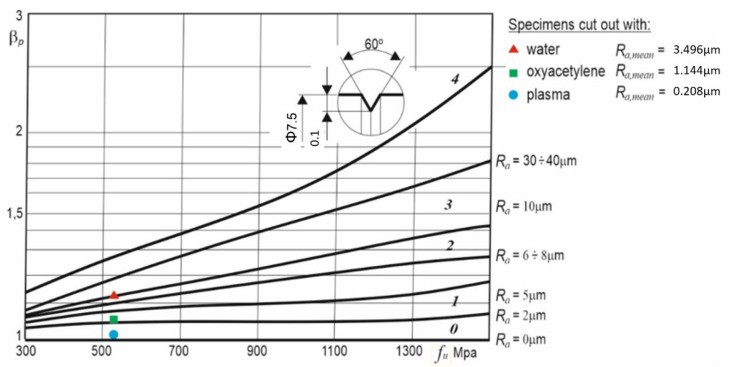
Effect of kind of machining on the value of surface condition coefficient β_p_ for tension or bending, depending on tensile strength of steel and kind of machining for: 0—polished, 1—ground, 2—fine rolled, 3—coarse rolled, and 4—sharply ring-notched (for comparison) specimens. Adapted with permission from Ref. [20], 2021, Wydawnictwo Naukowe PWN.

The effect of the kind of machining and the surface layer condition on the fatigue strength is expressed by surface condition coefficient β*_p_* as a ratio of the fatigue strength of an unnotched (polished) specimen to the latter’s strength after machining. The higher the surface condition coefficient, the lower the specimen’s fatigue strength due to surface irregularities. Figure 16 shows the results of experiments [20] in which the effect of the kind of machining (grinding, fine rolling, coarse rolling) on the value of coefficient β*_p_*, depending on the tensile strength, was studied. As one can see, the surface condition coefficient increases with surface roughness. Additionally, the mean surface roughness values of the faces of cut of in-house specimens of type A, B, and C for steel S355J2N with tensile strength *f_u_* = 510 MPa were included in the figure.

Table 8 contains the measured values of β*_p_* (according to Figure 16) and the calculated fatigue categories Δ*σ_c_* for each of the cutting technologies and the percentage differences relative to the results for the specimens cut out with plasma. Judging by the differences, the condition of the surface of the specimens has a relatively small (up to 12%) effect on their fatigue strength in comparison with the technologies used to cut them (55%).

The fatigue strength of the specimens cut out with oxyacetylene (Δ*σ_c_* = 226 MPa) is higher than that of the specimens cut out with plasma (Δ*σ_c_* = 146 MPa) even though the surface roughness after cutting with plasma is smaller than in the case of the other cutting technology. This is due to the significant effect of material hardening in the heat-affected zones. In both cases, acicular structures (Figure 12) with comparable heat-affected zone depths measured from the surface of the cut were obtained. However, in the case of plasma cutting, the hardness measured at the cut edge (310 HV10) was 27% greater than for the specimens cut out using the gas cutting technology (244 HV10). A similar agreement between the results was obtained in the tests described in the work of [18], where the specimens cut out with plasma were characterized by the highest hardness (280 HV10) and lower fatigue strength (Δ*σ_c_* = 239 MPa) in comparison with the oxygen cut specimens for which hardness amounted to 190 HV10 and fatigue strength to 264 MPa.

The results of the comparative tests indicate that the gas cutting technology used so far to cut out connectors for the innovative composite dowel joint is more advantageous than the plasma cutting technology or the water cutting technology. Furthermore, oxyacetylene cutting is the cheapest and most available cutting technology in prefabrication plants.

## 5. Conclusions

From the results of the fatigue tests carried out on steel S355J2N specimens cut out using different cutting methods, i.e., plasma cutting, water jet cutting, and oxyacetylene cutting, the following conclusions, providing a basis for further analyses leading to the development of design guidelines for steel connectors of the composite dowel type, can be drawn:

1. The technology of cutting out dowels of the composite dowel type has a bearing on their fatigue strength. Connectors cut out using oxyacetylene cutting can have higher fatigue strength than the ones cut out using plasma cutting or water jet cutting;
2. The effect of the technology used to cut out steel connectors of the composite dowel type can be greater than that of the condition of the face of the cut;
3. The slopes of the fatigue strength curves determined for the cut-out specimens: *m_A_* = 10 for water jet cutting, *m_B_* = 17 for oxygen cutting, and *m_C_* = 8 for plasma cutting, corroborate the conservative standard recommendation *m = 3* according to the work of [16];
4. The FAT125 fatigue curve according to the work of [16] can be appropriate for the design of composite dowel connectors to be cut out using oxygen cutting, plasma cutting, and water jet cutting. Nevertheless, further experimental studies (the S-N curve method) need to be carried out on beam specimens of composite structures in order to verify the fatigue curve for the composite dowel connector.

## Figures and Tables

**Table 1:** Test stress ranges Δ*σ_i_* for cutting technologies.

Specimen Type	Δ*σ* (MPa)	Specimen Type	Δ*σ* (MPa)	Specimen Type	Δ*σ* (MPa)
A Water jet cutting	150	B Oxyacetylene cutting	200	C Plasma cutting	125
	175		225		150
	200		250		175
	300		300		200
	325		-		225
	350		-		-

**Table 2:** Test results for specimens A.

Specimen No. (-)	*F* (kN)	Δ*σ* (MPa)	*N_f_* (Cycles)
12A	30	150	5,402,341
13A			5,467,028
18A			5,034,480
11A	35	175	779,609
16A			1,209,227
17A			2,744,983
14A	40	200	536,918
15A			336,773
1A	60	300	7619
2A			9174
3A			8339
7A	65	325	3722
8A			3330
9A			3339
5A	70 70	350 350	2175
6A			2225
4A			2129
22A			1225

**Table 3:** Test results for specimens B.

Specimen No. (-)	*F* (kN)	Δ*σ* (MPa)	*N_f_* (Cycles)
13B	40	200	5,476,832
14B			3,463,974
8B	45	225	5,249,668
9B			3,475,725
10B			3,568,974
11B			5,882,781
12B			2,870,595
3B	50	250	338,745
4B			1,030,958
5B	60	300	13,463
6B			32,353
7B			15,718

**Table 4:** Test results for specimens C.

Specimen No. (-)	*F* (kN)	Δ*σ* (MPa)	*N_f_* (Cycles)
12C	25	125	5,000,000
13C			5,000,000
14C			5,000,000
7C	30	150	5,000,000
10C			1,714,866
11C			430,863
3C	35	175	1,143,120
5C			444,094
6C			716,537
9C	40	200	196,487
2C	45	225	141,694
4C			70,782
8C			108,665

**Table 5:** Values of Δ*σ_c_* and *m* and regression curves for considered cutting technologies.

Cutting Technology	Regression Curve Equation	Fatigue Category Δ*σ_c_* (MPa)	Fatigue Curve Slope *m*
water A	Δ*σ =* 764.6*∙(N)*^−0.105^	167	10
oxyacetylene B	Δ*σ =* 531.6*∙(N)*^−0.059^	226	17
plasma C	Δ*σ =* 990.6*∙(N)*^−0.132^	146	8

**Table 6:** Results of roughness measurements: *R_a_*—roughness, *R_a,mean_*—mean roughness.

Specimen No. (-)	*R_a_* (μm)		*R_a,mean_* (μm)
	L	P	
2A	3.358	3.529	3.496
14A	3.889	3.311	
17A	3.715	3.172	
4B	1.531	1.719	1.444
10B	1.158	1.166	
14CB	1.530	1.561	
6C	0.296	0.369	0.208
8C	0.314	0.269	
10C	0.355	0.329	

**Table 7:** Depth of cut heat-affected zone (CHAZ).

Specimen No. (-)	CHAZ (μm)
4B	450
10B	550
14B	550
6C	505
8C	517
10C	475

**Table 8:** Values of β*_p_* and Δ*σ_c_* and differences between results for tested specimens.

Cutting Technology	*β_p_* (-)	Difference between *β_p_* Results (%)	Δ*σ_c_* (MPa)	Difference between Δ*σ_c_* Results (%)
Water (A)	1.13	12	167	14
Oxyacetylene (B)	1.05	4	226	55
Plasma (C)	1.01	0	146	0

## Data Availability

Data sharing not applicable.

## Abbreviations

The following symbols and notations, in the order in which they appear in the text, are used in this paper:

CHAZ	Cut heat-affected zone
R	Stress ratio
∆σ	Nominal stress range
F	Force
N_f_	Number of cycles to failure
logA	Intercept of mean S-N curve
m	Slope of fatigue strength curve
R_a_	Surface roughness
β_p_	Coefficient of surface condition

The other symbols used in this paper are explained when they appear in the text for the first time.
